# A Good Book on Massage

**Published:** 1912-02-10

**Authors:** A. E. Davis

**Affiliations:** Park Farm, Wallingford


*To the Editor of* The Hospital.

### A GOOD BOOK ON MASSAGE.

*To the Editor of* The Hospital.

Sir,—Will you in your next issue give the name of a good book on massage?

Yours truly,
(Nurse) A. E. Davis.

Park Farm, Wallingford, February 5.

[Mrs. C. Hale, "Art of Massage" (6s. post free) and Miss M. Atkey, "Massage in Practice" (2s. 9d. post free) (which is intended more for certificated workers) are two admirable books, and are published by and can be obtained from The Scientific Press, Ltd., 28 Southampton Street, Strand, W.C.—Ed. The Hospital.]

Considerations of space have obliged us to hold over a letter from Mr. Edward Warren, F.R.I.B.A., on the additions to Radcliffe Infirmary.

